# 2397. Infective Endocarditis is Associated With Higher Mortality and Worse Clinical Outcomes in Patients Hospitalized with a Concomitant Diagnosis of Heart Failure

**DOI:** 10.1093/ofid/ofad500.2017

**Published:** 2023-11-27

**Authors:** Deepali Boothankad Sharath, Leonid Khokhlov, Michael Fatuyi, Anar Patel

**Affiliations:** TriHealth Good Samaritan Hospital, Cincinnati, Ohio; TriHealth Good Samaritan Hospital, Cincinnati, Ohio; TriHealth Good Samaritan Hospital, Cincinnati, Ohio; TriHealth Infectious Diseases, Cincinnati, Ohio

## Abstract

**Background:**

The United States has the highest incidence of infective endocarditis (IE) in the world at 15 per 100,000 people with increasing incidence due to the aging population and growing rates of intravenous drug use (IVDU). Heart failure (HF) is the most common complication and the main cause of death in patients with IE. There is limited evidence on the clinical outcomes in IE populations affected by HF. We sought to investigate the demographics and outcomes of this cohort of patients.

**Methods:**

The Nationwide Inpatient Sample (NIS) database was queried between 2017-2020 for adult patients who were hospitalized with the primary diagnosis of IE. The secondary diagnosis was HF. The primary outcome evaluated was inpatient mortality. The secondary outcomes were severe sepsis, septic shock, cardiogenic shock, cardiac arrest, invasive mechanical ventilation, length of stay (LOS), and total hospital charge. Multivariable logistic and Poisson regression analyses were used to estimate clinical outcomes. P-value < 0.05 was significant.

**Results:**

We identified a total of 250,070 hospitalizations with the diagnosis of IE in 2017-2020, 95,320 of which also had HF (38%), 45% of which with specified systolic heart failure. Patient characteristics are shown in Table 1. Patients admitted with IE and concurrent HF had significantly higher inpatient mortality and worse clinical outcomes. (Table 2 and Figure 1).

**Figure:**
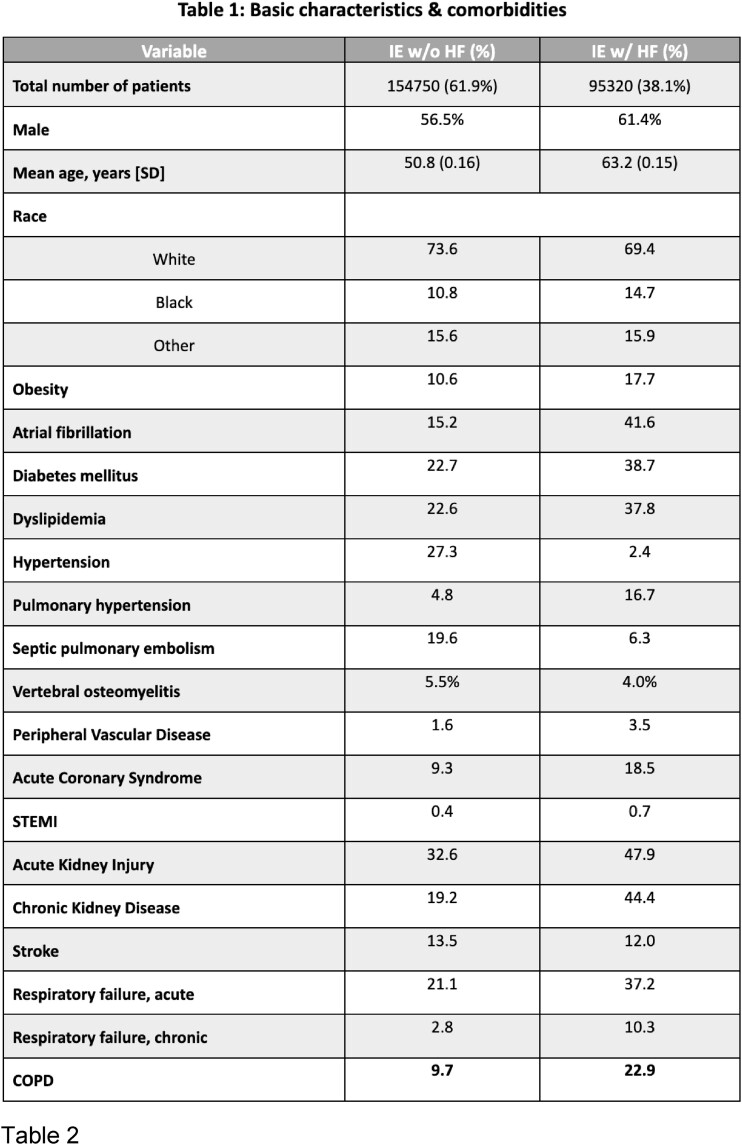

**Figure:**
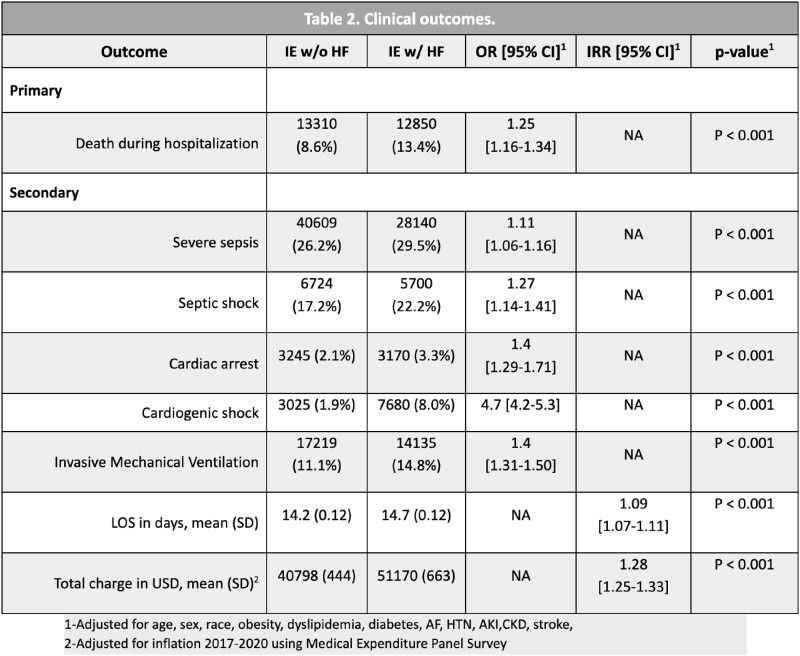

Figure 1

**Figure:**
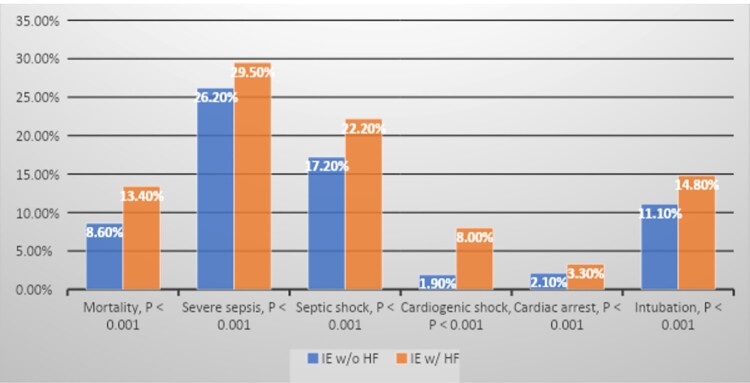

Clinical Outcomes in Patients with IE and Concomitant HF

**Conclusion:**

Our study showed that patients with IE and concurrent HF tended to be older males, Caucasians, and obese. This cohort was more likely to have atrial fibrillation, diabetes mellitus, dyslipidemia, acute coronary syndrome, ST elevation MI, acute renal failure, and chronic kidney disease (Table 1). Hence, cardiac dysfunction in IE patients is associated with renal and pulmonary impairment, higher rates of infectious complications, and a more severe hospitalization course. This can be explained by the myocardial dysfunction that predisposes to end-organ damage. When managing patients with IE and co-existing HF, clinicians should be aware of this predictor of negative outcomes. Further studies are necessary to describe the more recent trends given better antibiotic therapy, control of IV drug use, and earlier surgical interventions.

**Disclosures:**

**All Authors**: No reported disclosures

